# Predictors of preoperative cognitive dysfunction in adults with Moyamoya disease: a preliminary research

**DOI:** 10.1186/s12883-021-02511-2

**Published:** 2022-01-06

**Authors:** Jian Sun, Zhiyong Shi, Lebao Yu, Yujie Wen, Dong Zhang

**Affiliations:** 1 Department of Neurosurgery, Beijing Tiantan Hospital, Capital Medical University, 119 South Fourth Ring West Road, Fengtai District, Beijing, 100070 MN China; 2 Department of Neurosurgery, Beijing Changping District Hospital, Beijing, China; 3 Department of Neurosurgery, Nanjing Drum Tower Hospital, The Affiliated Hospital of Nanjing University Medical School, Nanjing, Jiangsu China; 4 Department of Psychiatry, The National Clinical Research Center for Mental Disorders & Beijing Key Laboratory of Mental Disorders & Beijing Institute for Brain Disorders Center of Schizophrenia, Beijing Anding Hospital, Beijing, China

**Keywords:** Moyamoya disease (MMD), Cognitive dysfunction, Digital subtraction angiography (DSA), Moyamoya vessels, Predictors, Risk factors, Vascular disorder

## Abstract

**Objective:**

To explore potential risk factors of preoperative cognitive dysfunction in adult patients with moyamoya disease (MMD) and discuss significance of moyamoya vessels.

**Methods:**

The author reviewed adult MMD patients harboring no parenchymal infarction or hemorrhage underwent a standardized neuropsychological assessment test battery from December 2018 to May 2019. The authors defined patients with cognitive dysfunction as cognitive impairment shown on 3 or more neuropsychological tests. According to the presence of cerebral angiography, arterial stenosis, moyamoya vessels, and compensatory arteries were conducted. Univariate and multivariate analyses were performed to identify predictors for cognitive dysfunction before surgery. Subgroup analyses by onset type and Suzuki stage were carried out to identify specific predictors for preoperative cognitive dysfunction.

**Results:**

In total, 29 of 92 (31.52%) patients had cognitive dysfunction. Multivariate analysis showed that moyamoya vessels generating from left hemisphere was recognized as independent predictor for cognitive dysfunction (*P* = 0.025, OR [95%CI], 0.085 [0.012–0.874]). For patients in left ICA-moyamoya subgroup, 19 of 45 (42.22%) cases with sparse moyamoya vessels had cognitive dysfunction (*P* = 0.031), while 22 (91.67%) of patients with dense moyamoya vessels had normal cognition (*P* = 0.004). Moyamoya vessels arising from ophthalmic artery had no significant association with cognitive dysfunction (*P* = 0.111). Multivariate analysis found that moyamoya vessels originating from left ICA was recognized as independent predictors for preoperative cognitive dysfunction (*P* = 0.048, OR [95%CI], 0.394 [0.132–0.926]).

**Conclusions:**

Moyamoya vessels arising from left hemisphere was a risk factor for the preoperative cognitive dysfunction in adult patients with MMD, with the denser moyamoya vessels, the less cognitive dysfunction. The current study offers a new perspective of moyamoya vessels and supporting data for choosing MMD candidates on cerebral revascularization.

## Introduction

Moyamoya disease (MMD) was a relatively rare cerebrovascular disease with unknown etiology, characterized by progressive stenosis of internal carotid artery (ICA) termination and the formation of smoke-like vessels at the base of brain [1–3]. The brain was in a state of ischemia and hypoxia resulting in the formation of moyamoya vessels due to the natural progress of MMD [4]. Zhao et al. reported that moyamoya vessels played a radiologic biomarker for neovascularization after indirect bypass [5]. The underlying pathophysiological mechanisms and potential effect of moyamoya vessels remained unknown.

Previous literature reported that cognitive function of adult patients with MMD were often impaired due to insufficient cerebral blood flow perfusion and hypoxia [6–10]. About 1/3–2/3 adult MMD patients had cognitive impairment, but the degree was moderate [7, 10–12]. So far, cognitive dysfunction was evaluated with perfusion assessment according to previous published reports, such as perfusion CT (CTP), perfusion positron emission tomography (PET), single photon emission computed tomography (SPECT), and even functional magnetic resonance imaging (fMRI) [13–15]. However, digital subtraction angiography (DSA) was also a useful tool for estimation of brain collateral circulation, which had never been used to evaluate cognitive impairment in adult patients with MMD in previous literature. The keynote of this research was to study potential risk factors of preoperative cognitive dysfunction in adult patients with MMD based on DSA findings, and discuss significance of moyamoya vessels.

## Methods

**Fig. 1:**
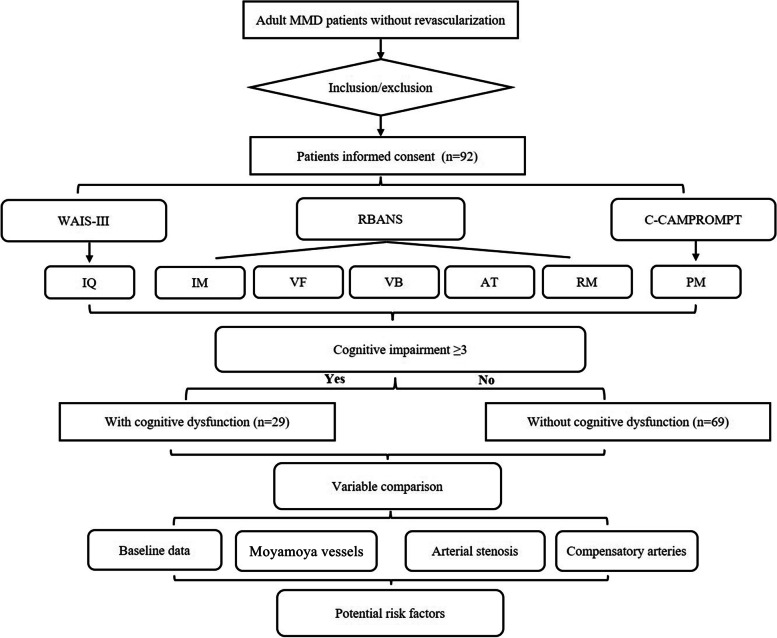
Flow chart of this research sample. IQ = intelligence quotient, IM = immediate memory, VF = verbal fluency, VB = visual breadth, AT = attention, RM = retrospective memory, PM = prospective memory, WAIS-III=Wechsler Adult Intelligence Scale Version 3, C-CAMPROMPT = the Chinese Cambridge Prospective Memory Test, RBANS = repeatable battery for the assessment of neuropsychological status

The study was approved by the ethics committee of Beijing Tiantan Hospital and written informed consent was obtained from all patients on admission. The ethics number was KY 2019–084-02. The criteria for admission were: (1) Patients were diagnosed with MMD by digital subtraction angiography according to the guideline for MMD (criteria of the Research Committee on Spontaneous Occlusion of the Circle of Willis, 2012) [16], (2) Patients older than 18 and younger than 70 years old, (3) Patients without paralysis or aphasia, (4) Patients without infarction or hemorrhage on CT or MRI images, (5) Patients without psychiatric disorders, (6) patients with right-handedness, (7) Patients performed with cerebral angiography and cognitive function test battery before revascularization surgery. Patients with the diagnosis of moyamoya syndrome (MMS), presence of posterior cerebral artery (PCA) involvement, and patients did not satisfy this inclusion were ruled out from the study. A consecutive of 92 adult patients with MMD from December 2018 to May 2019 were enrolled in this study. This study is complicated due to series of cognitive battery tests involved, and a flow chart was designed for easily understood (Fig. 1).

### Cognitive function testing procedure [17]

Cognitive function test battery included Wechsler Adult Intelligence Scale Version 3 (WAIS-III), the Chinese Cambridge Prospective Memory Test (C-CAMPROMPT), and repeatable battery for the assessment of neuropsychological status (RBANS) scale.

### WAIS-III Test [18]

This test was divided into four parts, including general knowledge (GK), similarity (SI), visual puzzle (VP), and block design (BD). The scores obtained were converted into standard scores, and transformed into intelligence quotient (IQ).

### RBANS test [19]

This test included five parts, including immediate memory (IM), verbal fluency (VF), visual breadth (VB), attention (AT) and retrospective memory (RM). IM included list learning and story memory tests. VB included picture copy and line orientation tests. VF included picture naming and semantic fluency tests. AT included digital breadth and coding tests. RM included sum of list/story/figure and list recognition. All the testing scores of RBANS were original scores, which needed to be converted to scale scores for comparison among individuals.

### C-CAMPROMPT [20]

Prospective memory (PM) test included time-based prospective memory (TBPM) and event-based prospective memory (EBPM). Adult patients with MMD were asked to perform several tasks including time-based tasks and event-based tasks during testing procedure. Total score of PM was the sum of scores of TBPM and EBPM.

### Definition of cognitive dysfunction

All raw data was shown as mean and standard deviations (SDs). According to published normative data, the raw scores of participants were converted into z scores adjusted for age, sex, and education level. For all tests, mild impairment was defined as a z score between 1.7SDs and 2.49SDs lower than normative mean, and severe impairment was defined as a z score ≥ 2.5 SDs below the normative mean [21]. Due to high numbers of our cognitive function tests, the authors defined patients with cognitive dysfunction as cognitive impairment shown on 3 or more neuropsychological tests.

### Scores and grades of DSA findings

According to the ROC analysis, the cut-off value of scores in moyamoya vessels, intracranial arterial stenosis, and extra−/intracranial compensatory artery numbers were calculated.

**Fig. 2:**
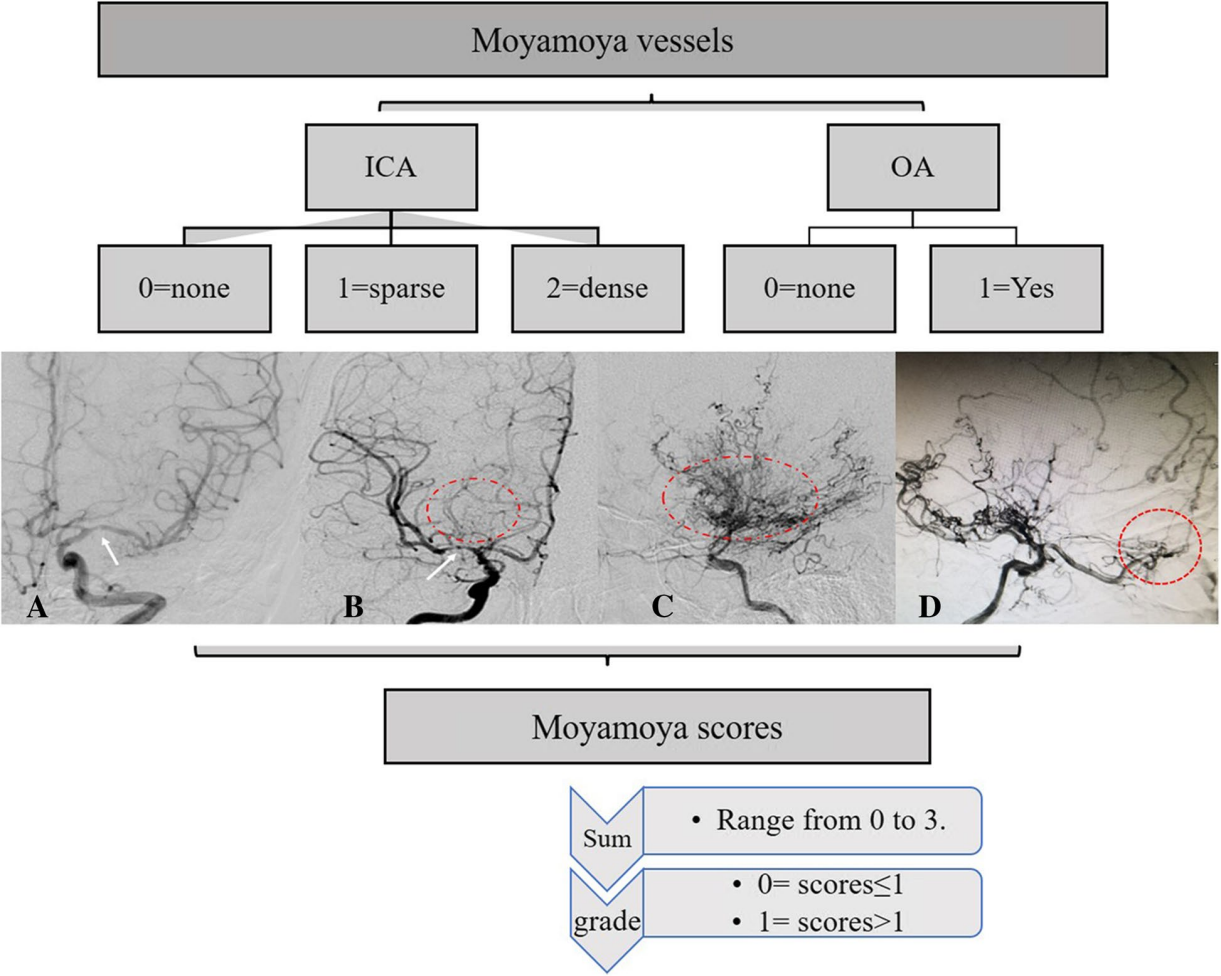
The diagram of scores and grades of moyamoya vessels. **A**-**C** Existence of “none”, “sparse” and “dense” moyamoya vessels arising from ICA (red circle). **D** moyamoya vessels originating from OA (red circle)

**Fig. 3:**
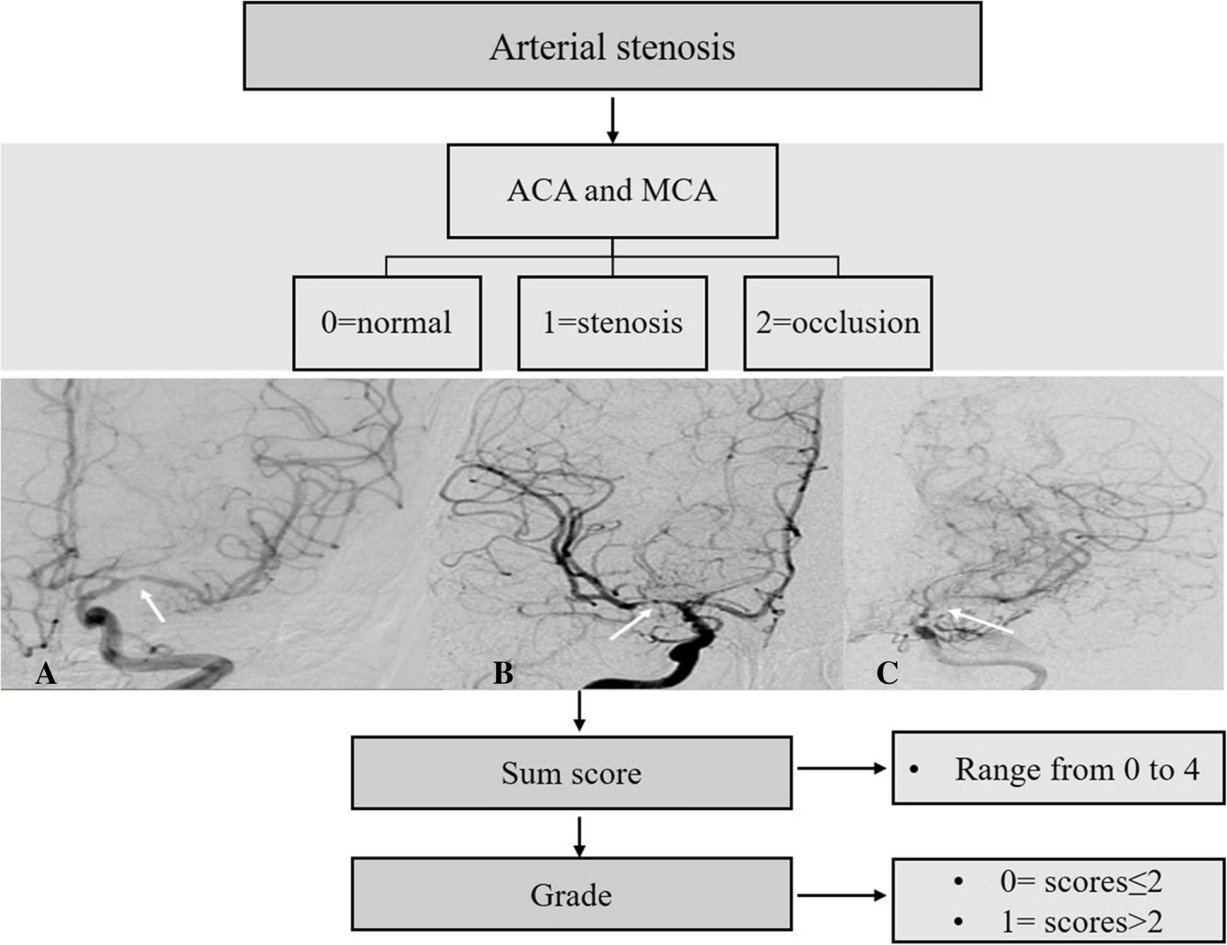
The diagram of scores and grades of arterial stenosis. A-**C** Mild stenosis, severe stenosis, and arterial occlusion of MCA (white arrow)

**Fig. 4:**
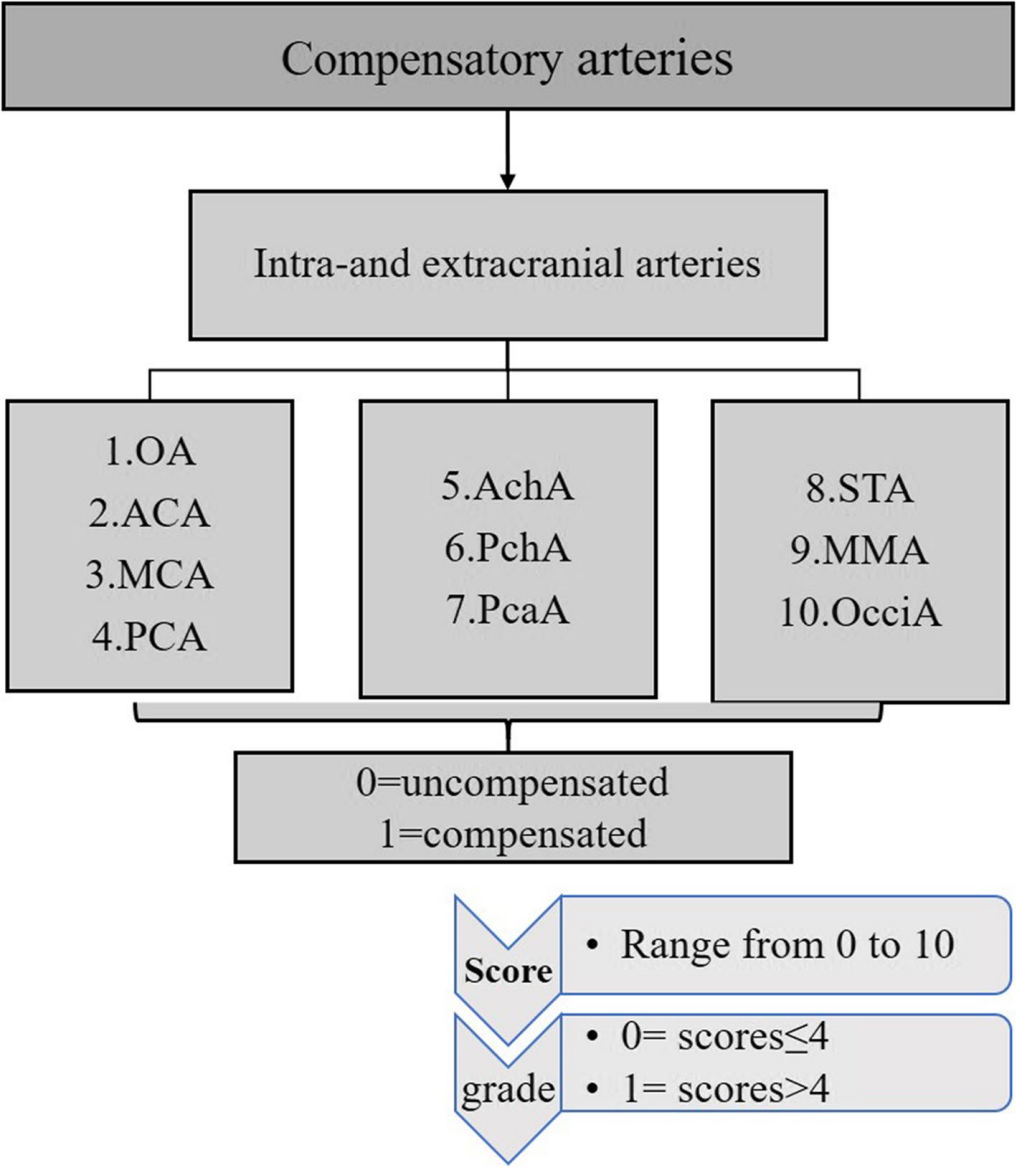
The diagram of scores and grades of extracranial and intracranial compensatory arteries

1. moyamoya vessel [5]**.**
  1. moyamoya vessel from ICA (moyamoya-ICA). Moyamoya-ICA could be divided into three grades: score 0 (none), no obvious moyamoya vessels; score 1 (sparse), moyamoya vessels formation at the base of brain, but sparser; score 3 (dense), large number of moyamoya vessels anastomosed to form a network at the end of the ICA and expanded in all directions at the base of brain.
  2. moyamoya vessels from ophthalmic artery (moyamoya-OA). Moyamoya-OA could be divided into two grades and score: score 0 was no moyamoya vessels formation, and score 1 was the existence of moyamoya vessels.
  3. The sum scores of moyamoya-ICA and moyamoya-OA varied from 0 to 3 points. The author artificially defined that grade 0 was the score ≤ 1 indicating no moyamoya vessels or sparse moyamoya vessels, and grade 1 was the score ≥ 2 representing the dense vasculature of moyamoya vessels (Fig. 2).
2. Intracranial artery stenosis.
3. Based on stenosis of anterior cerebral artery (ACA) and MCA, intracranial arterial stenosis could be divided into three grades: score 0 (normal), ACA or MCA was approximately normal or even dilated; score 1 (stenosis), the origin of ACA or MCA origin had stenosis, but distal vessels could still be seen through more contrast agents; score 2 (occlusion), occlusion at the proximal portion of ACA or MCA, with little or no contrast agent passing through the distal vessels.
4. The sum of ACA (0–2) and MCA (0–2) ranged from 0 to 4 points. The author artificially defined that grade 0 was stenosis scores≤2 indicating normal or mild stenosis, whereas grade 1 was scores ≥3, which represented the existence of severe stenosis or occlusion (Fig. 3).
5. Intra−/extracranial compensatory artery numbers.

- A total of 10 arteries were evaluated including OA, ACA, MCA, anterior choroidal artery (AchA), posterior choroidal artery (PchA), peri-callous artery (PcaA) and posterior cerebral artery (PCA), superficial temporal artery (STA), middle meningeal artery (MMA) and occipital artery (OcciA). For each artery, score 0 was the artery was un-compensated and score 1 was compensated.
- The sum of scores for compensation theoretically ranged from 0 to 10. Grade 0 was compensation score ≤ 4, which represented fewer compensatory vessels. Grade 1 was compensation score ≥ 5, which represented more compensatory vessels (Fig. 4).

### Statistical analysis

1. Univariate analysis. Continuous variables in our study were analyzed by t-test, including age and education time. Categorical variables were analyzed by chi-square test. For categorized variables, if any value was less than 5, Fisher exact test is used for analysis. The rank variables were analyzed by Mann-Whitney test. For rank variables with statistical significance, post hoc multiple comparisons and Bonferroni correction were used. The *P* value < 0.016 (0.5/3) had statistical significance.
2. Multivariate analysis: For the variables with significant statistical differences in univariate analysis (*P* < 0.05), and the factors that might have significant influence on cognitive function of adult patients with MMD according to our clinical experience, such as gender, age and education time, binary logistic regression with enter model was applied to multivariate analysis. The *P* value, the ratio of Odds (OR) and the 95% confidence interval (CI) of the ratio were calculated.

SPSS22.0 software (IBM Corp., Chicago, IL, USA) was used for statistical analysis. The significance level was set at P value< 0.05.

## Results

### Patient demographics

A total of 92 consecutive adult MMD patients (patient age range 18–62 years, mean 37 years) satisfied the inclusion criteria in this research; 28 cases (30.43%) had hemorrhagic symptoms, and 64 cases (69.56%) had ischemic symptoms. Forty-five females (48.91%) and 47 males (51.09%) were recruited. Adult MMD Patients with Suzuki stage of I, II, III, IV, V and VI were 5, 13, 21, 16, 26, 4 cases in the left hemisphere, and 17, 9, 13, 22, 27, 4 cases in the right hemisphere, respectively (Table 1). There was no significant difference in age, gender, education time, clinical type, and Suzuki stage of bilateral hemisphere between patients with normal cognition and patients with cognitive dysfunction (*P* > 0.05), except Suzuki stage II in left hemisphere (*P* = 0.021) (Table 2).

**Table 1:** Demographic data and clinical characteristics

Characteristic	Value	
No. of patients	92	
Age, yrs	37.65 ± 8.65	
Gender (female, n, %)	45 (48.91)	
Onset type (n, %)		
Ischemic	64 (69.56)	
Hemorrhagic	28 (30.43)	
Suzuki stage (n, %)	Left	Right
I	5 (5.43)	17 (18.48)
II	13 (14.13)	9 (9.78)
III	21 (22.83)	13 (14.13)
IV	16 (17.39)	22 (23.91)
V	26 (28.26)	27 (29.35)
VI	4 (4.35)	4 (4.35)

**Table 2:** Comparison of age, education time, gender and Suzuki stage in left or right hemispheres on cognitive dysfunction before surgery

Variables	Cognitive dysfunction		*P* value
	Normal (*n* = 63)	Dysfunction (*n* = 29)	
Age	37.32 ± 8.57	39.14 ± 8.79	0.516
Education time	13.87 ± 2.41	12.50 ± 3.94	0.244
Gender			
Male	28 (44.4%)	19 (65.5%)	0.075
Female	35 (55.6%)	10 (34.5%)	
Clinical type			
Ischemic	47 (74.6%)	17 (58.6%)	0.147
Hemorrhagic	16 (25.4%)	12 (41.4%)	
Suzuki stage (left)			0.208
I	3 (4.8%)	2 (6.9%)	0.649
II	5 (8.1%)	8 (27.6%)	0.021*
III	15 (24.2%)	6 (20.7%)	0.796
IV	11 (17.7%)	5 (17.2%)	0.615
V	26 (41.3%)	8 (27.6%)	0.206
VI	3 (4.8%)	1 (3.4%)	0.774
Suzuki stage (right)			0.555
I	11 (17.5%)	6 (20.7%)	0.711
II	5 (7.9%)	4 (13.8%)	0.456
III	8 (12.7%)	5 (17.2%)	0.539
IV	18 (28.6%)	4 (13.8%)	0.123
V	19 (30.2%)	8 (27.6%%)	0.801
VI	2 (3.2%)	2 (6.9%)	0.588

### Patient dysfunction characteristics

Of 92 adult patients with MMD, cognitive dysfunction was found in 29 patients (31.52%). Specifically, the numbers and percentage of cognitive impairment for adult MMD patients were 31 in IQ (33.69%), 34 in PM (36.96%), 16 in IM (17.39%), 1 in VB (1.09%),30 in VF (32.61%), 33 in AT (35.86%), and 18 in RM (19.56%), respectively (Table 3).

**Table 3:** Mean z scores and number of patients performing in the impaired range for each test

Tests	Mean z scores	No. of patients /impairment		
		Mild	Severe	Overall
no. of patients				92
IQ	−0.973	16	15	31 (33.69%)
PM	−1.35	13	21	34 (36.96%)
IM	−0.659	7	9	16 (17.39%)
VD	−0.244	1	0	1 (1.09%)
VF	−1.149	12	18	30 (32.61%)
AT	−1.484	12	21	33 (35.86%)
RM	−0.81	3	15	18 (19.56%)

*IQ* Intelligence Quotient, *PM* Prospective Memory, *IM* Immediate Memory, *VD* Visual Breadth, *VF* Verbal Fluency, *AT* Attention, *RM* Retrospective Memory

### Cognitive dysfunction and DSA findings evaluation

**Fig. 5:**
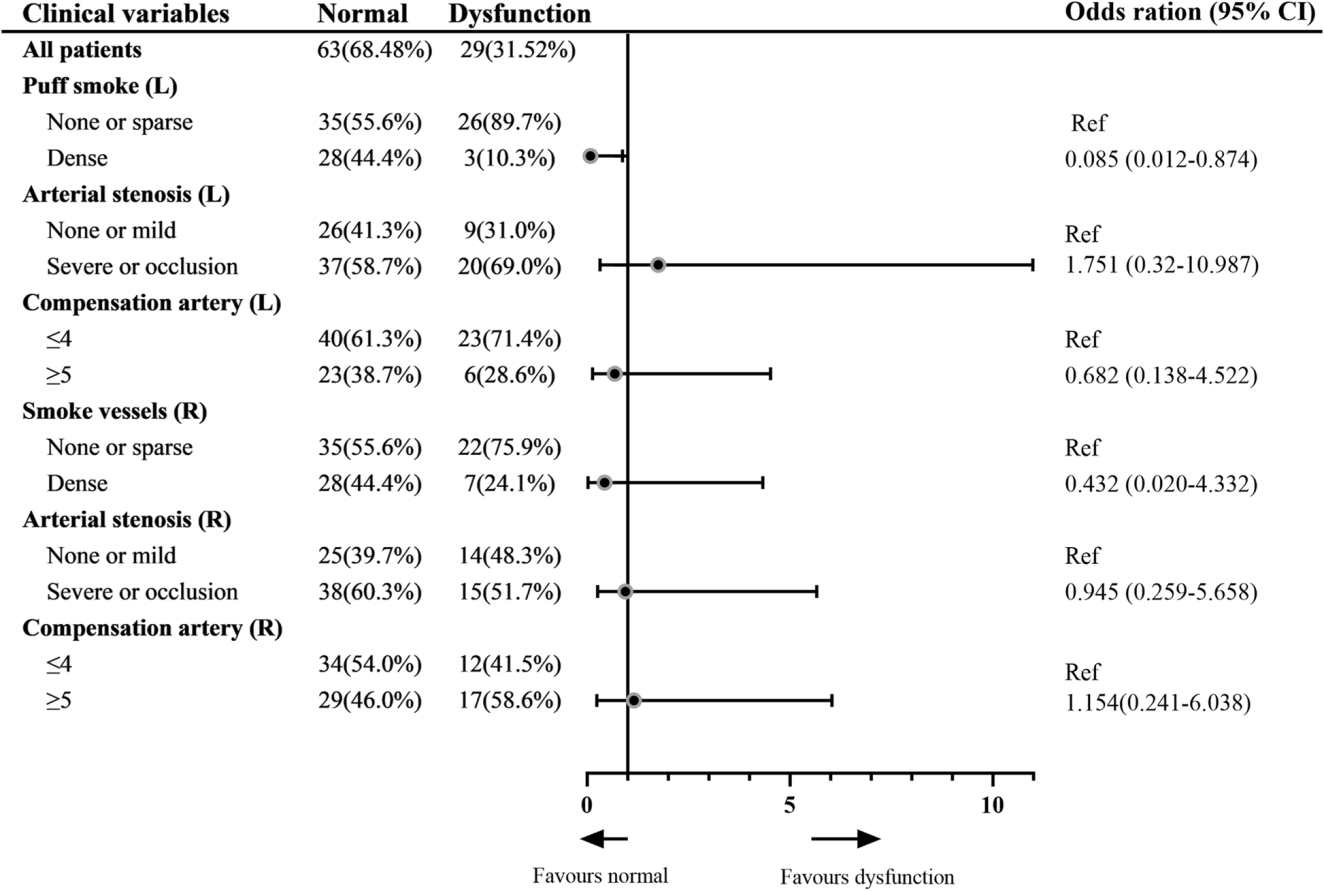
Univariate and multivariate analysis of bilateral moyamoya vessels, intracranial artery stenosis and intra-extracranial compensatory vessels on cognitive dysfunction in MMD before surgery. L = left, R = right

For moyamoya vessels arising from left hemisphere, 26 patients (89.7%) in none or sparse moyamoya group had cognitive dysfunction, which was significantly higher than 3 patients (10.3%) in dense group (*P* = 0.001). However, for moyamoya vessels originating from right hemisphere, 22 cases (75.9%) in none or sparse moyamoya group had cognitive dysfunction, which did not differ from 7 cases (24.1%) in dense group (*P* = 0.062). There was no difference in age, gender, clinical type, bilateral stenosis grade, bilateral compensation grade between patients with normal and cognitive dysfunction (*P*>0.05) (Table 2). To further rule out effects of various factors, multivariate analysis was conducted with the aforementioned significant factors or according to clinical experience. Moyamoya vessels generating from left hemisphere was recognized as independent predictors for cognitive dysfunction before surgery (*P* = 0.025, OR [95%CI], 0.085 [0.012–0.874]) (Fig. 5).

### Cognitive dysfunction and moyamoya vessels origin

To further distinguish effects of moyamoya vessels origin in left hemisphere, patients with the presence of moyamoya-ICA and moyamoya-OA was conducted. For patients in ICA-moyamoya subgroup, 19 of 45 (42.22%) cases with sparse moyamoya vessels had cognitive dysfunction (*P* = 0.031), while 22 (91.67%) of patients with dense moyamoya vessels had normal cognition (*P* = 0.004). However, cognitive dysfunction had no significant association with presence of moyamoya-OA (*P* = 0.111). Multivariate analysis was conducted with factors with the aforementioned significance, and moyamoya vessels originating from left ICA was recognized as independent predictors for preoperative cognitive dysfunction (*P* = 0.048, OR [95%CI], 0.394 [0.132–0.926]), especially for cases with dense moyamoya vasculature (*P* = 0.038, OR [95%CI], 0.159[0.004–0.860]) (Table 4).

**Table 4:** Univariate and multivariate analysis of moyamoya vessels in ICA or OA in left hemisphere on preoperative cognitive dysfunction

Moyamoya vessels	Cognitive dysfunction		Univariate analysis	Multivariate analysis	OR (95%CI)
	Normal (*n* = 63)	Dysfunction (*n* = 29)			
ICA			0.014*	0.048*	0.394 (0.132–0.926)
None	15 (23.8%)	8 (27.6%)	0.609_a_	0.084	Ref
Sparse	26 (41.3%)	19 (65.5%)	0.036_b_*	0.712	0.705 (0.110–4.519)
Dense	22 (34.9%)	2 (6.9%)	0.004_c_*	0.038	0.159 (0.004–0.860)
OA	21 (33.3%)	5 (17.2%)	0.111	0.343	0.324 (0.031–3.483)

*ICA* internal carotid artery, *OA* ophthalmic artery. Subscript “a”, “b”, and “c” represented the 2-group comparisons between none-sparse, none-dense, and sparse-dense moyamoya vessels, respectively. “*” *P* < 0.05

## Discussion

MMD was a group of chronic progressive disease characterized by formation of smoky vessels at the base of brain, which could result in cognitive impairment [10, 22–24]. Previous literatures reported that the proportion of cognitive impairment in patients with MMD was about 30% [25–27], which was consistent with our results. In this study, patients manifested cognitive impairment as IQ (33.69%), PM (36.96%), IM (17.39%), VD (1.09%), VF (32.61%), AT (35.86%), RM (19.56%), respectively. According to our knowledge, this is the study with largest number of reported cases and most comprehensive understanding of cognitive impairment in adult MMD patients so far, which is of great value for further research.

As is known that DSA was a commonly used clinical radiological examination for MMD, which could dynamically evaluate cerebral blood flow (CBF). Suzuki stage was a common and classical staging method for MMD [23, 28, 29]. In this study, we attempted to explore cognitive dysfunction in MMD with DSA, and no similar reports had been reported before. This study found that moyamoya vessels originating from left hemisphere in MMD was independent predictors for cognitive dysfunction before surgery (*P* = 0.025, OR [95%CI], 0.085 [0.012–0.874]). Cognitive dysfunction was rare for patients with dense moyamoya vessels, which included following situations: 1) both existence of moyamoya vessels originating from left OA and ICA concurrently; 2) dense moyamoya vessels originating from left ICA alone (*P* = 0.048, OR [95%CI], 0.394 [0.132–0.926]). It seemed that cognitive dysfunction occurred mostly in adult MMD patients with sparse moyamoya vessels, but rare for those with dense moyamoya vessels. Furthermore, we speculated that it was critical to preserve the function of left hemisphere (dominant hemisphere for the right-handedness) because of the existence of many functional linguistic areas for most people [30, 31]. In other words, the left was more preferrable for the MMD patients with uncertain surgical hemisphere, which was consistent with our clinical experience.

As is known that moyamoya vessels in patients with MMD were associated with cerebral ischemia / hypoxia, and played an important role in maintaining collateral circulation [32, 33]. Zhao Y reported that moyamoya vessels originating from the ICA had predictive effects on neovascularization in patients with MMD after indirect revascularization [5]. In our last paper, we also reported that moyamoya vessels could decrease perfusion of temporal and parietal lobe [34]. Based on our research, patients with dense moyamoya vessels had less cognitive dysfunction compared to patients with sparse moyamoya vessels. Therefore, in the case of progressive arterial stenosis and hemispheric ischemia, moyamoya vessels are of great value to maintain cerebral perfusion and preserve brain cognition. We hypothesized that smoky vascularization (moyamoya vessels) played a role in collateral circulation in MMD when suffering from cerebral ischemia and hypoxia, and adult MMD patients with relatively intact cognition were prone to gain cognitive benefits in case of recurrent stroke attack. C Tan. et proposed that moyamoya vessels for MMD were embryonic origin, caused by abnormalities of sprouting angiogenesis, vessel fusion, and pruning. They argued that so-called “moyamoya vessels” were unfused primitive vessels or capillary plexus. Tan believed that compensatory-undegenerating moyamoya vasculature played an important role in various stages of brain development [35]. Thus, potential of moyamoya vasculature is a signal of brain health. Adult MMD patients with dense moyamoya vessels might have high potential of neovascularization to maintain stability of brain perfusion for a long time, thereby retaining cognitive function of adult patients with MMD. This hypothesis needed to study and confirm further.

In addition, when adult patients with MMD seeking for help in outpatient with DSA data only, the clinician could estimate the cognitive function probably. Moyamoya vessels arising from left hemisphere was a signal of cognitive dysfunction for adult MMD patients preliminarily, with more cognition loss in “sparse” subgroup. Surgical revascularization was able to rescue remaining cognition in MMD with sparse moyamoya vessels. Moreover, there was no significant difference between cognitive dysfunction between intracranial arterial stenosis or compensatory artery numbers from intra−/extracranial, indicating that moyamoya vessels played a role in maintaining cerebral perfusion and preserving cognitive function in MMD. In the long run, that was to say spontaneous formation of compensatory arteries could not reverse cognitive dysfunction in adult MMD patient, which was caused by chronic ischemia due to progressive intracranial arterial stenosis. From the perspective of cognitive preservation, surgical revascularization was essential when necessary. Cognition loss dynamically evolved with the dynamic change of moyamoya vessels in left hemisphere. Surgical revascularization could promote neovascularization after surgery, which could reduce the possibility of cognition loss due to the dynamic evaluation of moyamoya vessels during the natural course in MMD. In further research, it was worthwhile to explore the relationship between the preoperative moyamoya vessel concentration and postoperative brain cognitive function, and which subgroup of moyamoya vessels benefited the most. Overall, we speculated that moyamoya vessels were signals of disease process, potential for neovascularization and reserve of brain cognition in MMD, which was of value to reconsider this directive factor of moyamoya vessels in conduct of the preoperative assessment.

The limitation of this study was as follows. First, the number of cases enrolled was limited. Subgroup analysis might result in statistical bias, and the sample size needed to be further expanded. Second, the inclusion criteria of our study were adult MMD patients without apparent infarction in cerebral cortex and basal ganglia, without the exclusion of lacunar infarction. However, lacunar infarction itself might have an impact on cognitive function of patients with MMD. Third, this study only explored the preoperative cognitive status of adult patients with MMD. However, status of postoperative cognitive function was not included. Moreover, the potential pathophysiological mechanism was still unknown, which needed to be further studied. Fourth. the confirmation of cognitive dysfunction was 3 or more cognitive impairments in IQ, PM, IM, VD, VF, AT and RM. Our study demonstrated that cognitive dysfunction could be identified for MMD with assessment of moyamoya vessels, while no specific cognitive impairment could be identified. Fifth, cognitive dysfunction assessment was not combined with brain perfusion examinations in this study. The mechanism underlying dynamic process of moyamoya vessels might be the hemispheric perfusion status, which would be addressed in combination with brain perfusion examination, such as CTP, SPECT, or fMRI. Sixth, CBF evaluation in adult MMD patients was a holistic and dynamic process. We artificially divided them into three parts: stenosis, compensation artery numbers and moyamoya vessels, which needed to be evaluated in combination with brain perfusion examination. Seventh, we concentrated on the number of arteries for the evaluation of compensation artery, without considering the area and extent of vascular compensation. Eighth, all adult MMD patients recruited into our study were right-handed, the cognitive function of left-handed were needed to explore further.

## Conclusions

Moyamoya vessels arising from left hemisphere was a risk factor of preoperative cognitive dysfunction for adult patients with MMD, with the denser moyamoya vessels, the less cognitive dysfunction. The current study offers a new perspective of moyamoya vessels and supporting data for choosing MMD candidates on cerebral revascularization.

## Data Availability

The datasets generated during and/or analyzed during the current study are available from the first author on reasonable request (Jian Sun, sjzqbx213@126.com, and Zhiyong Shi, szy1195156829@aliyun.com).
